# Evaluating Distribution and Prognostic Value of New Tumor-Infiltrating Lymphocytes in HCC Based on a scRNA-Seq Study With CIBERSORTx

**DOI:** 10.3389/fmed.2020.00451

**Published:** 2020-09-17

**Authors:** Lixing Li, Lu Shen, Jingsong Ma, Qiang Zhou, Mo Li, Hao Wu, Muyun Wei, Di Zhang, Ting Wang, Shengying Qin, Tonghai Xing

**Affiliations:** ^1^Department of General Surgery, School of Medicine, Shanghai General Hospital, Shanghai Jiao Tong University, Shanghai, China; ^2^Key Laboratory for the Genetics of Developmental and Neuropsychiatric Disorders (Ministry of Education), Bio-X Institutes, Shanghai Jiao Tong University, Shanghai, China; ^3^Institutes of Biomedical Sciences, Fudan University, Shanghai, China; ^4^Department of Liver Surgery, Liver Cancer Institute, Zhongshan Hospital, Fudan University, Shanghai, China; ^5^Key Laboratory of Carcinogenesis and Cancer Invasion (Ministry of Education), Fudan University, Shanghai, China

**Keywords:** HCC, TILs, immune risk score, scRNA-seq, CIBERSORTx, LASSO

## Abstract

Hepatocellular carcinoma (HCC) is a commonly diagnosed cancer with high mortality rates. The immune response plays an important role in the progression of HCC. Immunotherapies are becoming an increasingly promising tool for treating cancers. Advancements in scRNA-seq (single-cell RNA sequencing) have allowed us to identify new subsets in the immune microenvironment of HCC. Yet, distribution of these new cell types and their potential prognostic value in bulk samples from large cohorts remained unclear. This study aimed to investigate the tumor-infiltration and prognostic value of new cell subsets identified by a previous scRNA-seq study in a TCGA HCC cohort using CIBERSORTx, a machine learning method to estimate cell proportion and infer cell-type-specific gene expression profiles. We observed different distributions of tumor-infiltrating lymphocytes between tumor and normal cells. Among these, the CD4-GZMA cell subset showed association with prognosis (log-rank test, *p* < 0.05). We further analyzed CD4-GZMA cell specific gene expression with CIBERSORTx, and found 19 prognostic genes (univariable cox regression, *p* < 0.05). Finally, we applied Least absolute shrinkage and selection operator (LASSO) Cox regression to construct an immune risk score model and performed a prognostic assessment of our model in TCGA and ICGC cohorts. Taken together, the immune landscape in HCC bulk samples may be more complex than assumed, with heterogeneity and different tumor-infiltration relative to scRNA-seq results. Additionally, CD4-GZMA cells and their characteristics may yield therapeutic benefits in the immune treatment of HCC.

## Introduction

Liver cancer is the sixth most commonly diagnosed cancer and the fourth leading cause of cancer mortality worldwide (1). Hepatocellular carcinoma (HCC) accounts for highest proportion of primary liver cancers and can be caused by chronic hepatitis C virus (HCV) or hepatitis B virus (HBV), heavy alcohol drinking, and metabolic syndromes related to diabetes and obesity (2). Beyond traditional treatments, including surgical and loco-regional interventions, immunotherapy is emerging as a promising therapeutic tool to treat hepatocellular carcinoma (3). Yet, immunotherapies have made little progress in clinical practice and the characteristics of HCC tumors that may predict the response to immunotherapies remain largely unknown (4, 5).

Single-cell RNA sequencing (scRNA-seq) has allowed for comprehensive analysis of tissue microenvironments. A human liver cell atlas has been constructed by scRNA-seq sequencing, describing previously unknown subtypes of endothelial cells, Kupffer cells, and hepatocytes (6). New subsets of tumor-infiltrating lymphocytes (TILs) related to HCC have been identified, such as exhausted CD8^+^ T cells, exhausted Tregs, LAMP3^+^ dendritic cells (DCs), and tumor-associated macrophages (TAMs), gradually unraveling the immune landscape of hepatocellular carcinoma (7, 8). Though scRNA-seq technique is a powerful method resolving cellular heterogeneity, it remains impractical for large-scale analyses (9).

Based on the previous approach, CIBERSORT, that enables estimation of cell type abundances from bulk tissue transcriptomes, CIBERSORTx is able to infer cell-type-specific gene expression profiles and allow the use of single-cell RNA-sequencing data for large-scale tissue dissection (10–12).

**Figure 1:**
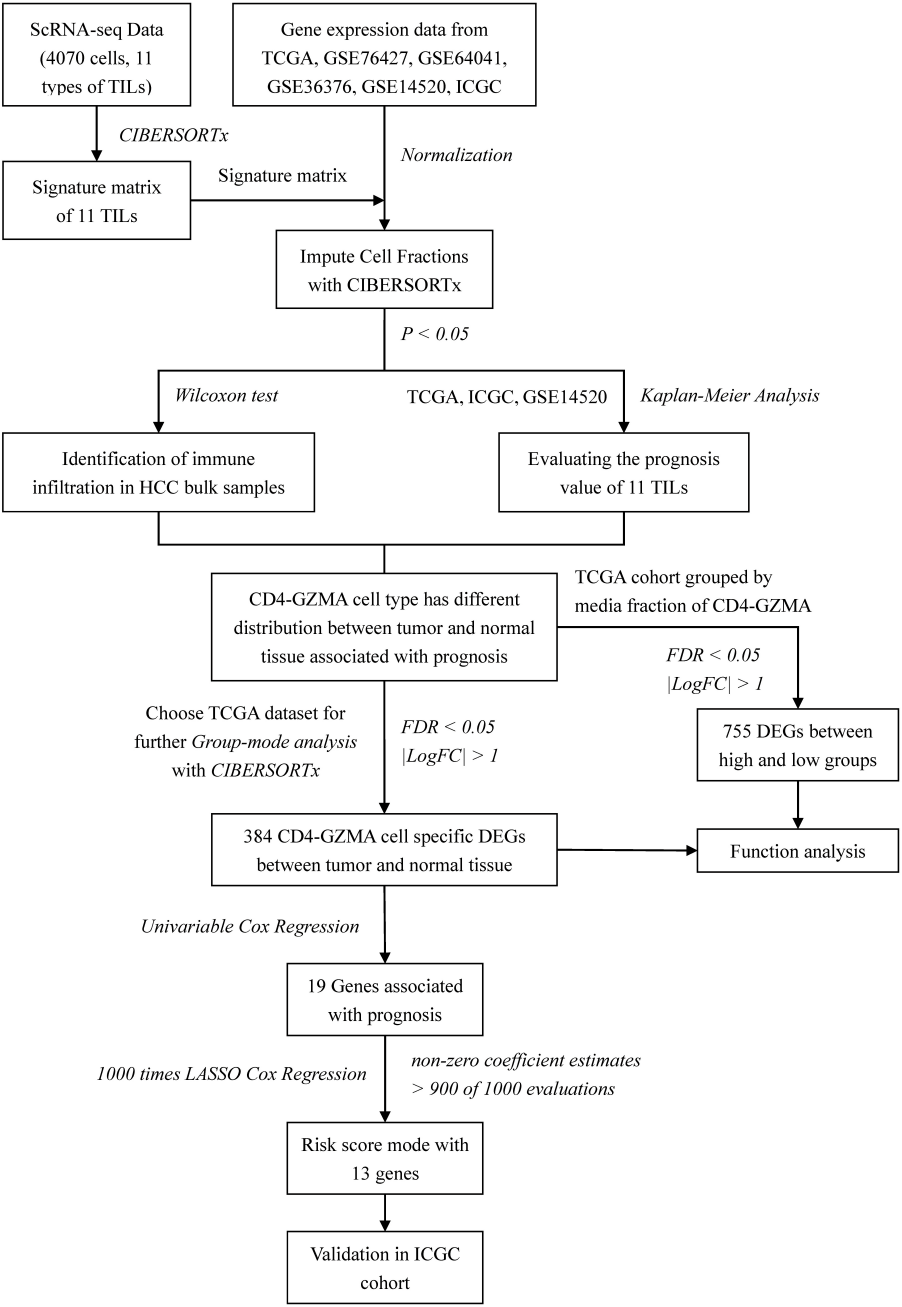
Flow chart of this study.

Several studies have explored the tumor microenvironments of HCC and assessed TILs for their overall survival (13–16). However, previous studies mostly selected mature molecular markers for common immune cells' identification and rarely focused on tissue-specific infiltered cell subsets. The scheme of our work was shown in Figure 1. In this study, we applied CIBERSORTx algorithm to realize combination analysis of scRNA-seq data of TILs in HCC and liver cancer gene expression profiles from TCGA. We explored the distribution and prognostic value of the TIL subsets. Importantly, we analyzed cell-type-specific gene expression profiles of one subset closely related to clinical outcome with CIBERSORTx (11, 12). In combination with univariate Cox regression analysis, least absolute shrinkage and selection operator (LASSO) Cox regression analysis was used to construct an immune risk score model from differentially expressed genes of cell-type-specific gene expression profiles, offering a significantly powerful means of predicting the prognosis of patients with HCC cancer.

## Materials and Methods

### Data

We obtained the transcripts per million (TPM) data of 5,063 cell samples with single-cell transcriptome profiling from GSE 98638 via the Gene Expression Omnibus (GEO) database (https://www.ncbi.nlm.nih.gov/geo/). The immune cells without a cell phenotype were excluded and 4,070 cells were finally left. The residual cells were all profiled by Smart-seq2 protocol and sequenced on Illumina HiSeq2500 and HiSeq4000. We downloaded an RNA-seq dataset of 269 HCC patients from TCGA (https://portal.gdc.cancer.gov/) and 229 patients from the ICGC database (https://icgc.org/). Clinical data including age, gender, TNM stage, follow-up time, and vital status were also collected. Inclusion criteria were: (1) pathology confirmed HCC, (2) clinical data from the patients were available, and (3) follow-up time >30 days. Two RNA-seq datasets of fragments per kilobases per million (FPKM) values were converted to transcripts per million (TPM) values in R. They were annotated by R package “org.Hs.eg.db.” The RNA-seq dataset of counts in TCGA was also downloaded for further analysis. The RNA-seq dataset were divided into tumor and normal groups. We also downloaded a normalized gene expression matrix of datasets GSE76427, GSE64041, GSE36376, and GSE14520 from the Gene Expression Omnibus (GEO) database (https://www.ncbi.nlm.nih.gov/geo/). They were annotated by R package “illuminaHumanv4.db,” “hugene10sttranscriptcluster.db,” or relative platform annotation files. Clinical data meeting inclusion criteria of GSE14520, including age, gender, TNM stage, follow-up time, and vital status, were also collected.

### Building the scRNA-Seq Signature Matrix

CIBERSORTx online analysis platform (https://cibersortx.stanford.edu/) was applied to infer cell-type-specific gene expression profiles without physical cell isolation. We first prepared and uploaded the single-cell expression matrix according to the instructions with CIBERSORTx. The default parameters remained. Then we ran “CIBERSORTx” and obtained a signature matrix of 11 cell types from scRNA-seq data.

### Impute Cell Fractions With CIBERSORTx

We prepared and uploaded the mixture datasets of tumor and normal groups obtained from TCGA, ICGC, GSE76427, GSE64041, GSE36376, and GSE14520 according to the instructions with CIBERSORTx. Then we chose the signature matrix we obtained before. Since scRNA data was derived from Smart-seq2, we selected “B-mode” to batch correction. We set permutations to 1,000. Other parameters retained the default. After running “CIBERSORTx,” we obtained the relative proportions of 11 subsets of tumor-infiltrating immune cells in each sample with *p*-value measuring the confidence of the results for the deconvolution. Samples with *P* < 0.05 were included in a further study.

### Differentially Expressed Gene Analysis

We analyzed the RNA-Seq data of counts for all 269 HCC patients obtained from TCGA. Patients were grouped into high and low groups by median of CD4-GZMA cell proportion. The analysis was performed using package “DESeq-2.” Setting the cut-off criteria as |log2 fold change| > 1.0 and adj.p <0.05, we identified 755 differentially expressed genes (DEGs).

### Enrichment Analysis of DEGs

The Gene Ontology Resource (http://geneontology.org/) and KOBAS 3.0 (http://kobas.cbi.pku.edu.cn/kobas3) (17) were applied for GO analysis of BP term and pathway enrichment. An adjusted *p* < 0.05 was set as the cut-off.

### Impute Cell-Type-Specific Gene Expression With CIBERSORTx

We run CIBERSORTx group-mode to impute cell type-specific gene expression on tumor and normal classes from TCGA HCC patients separately. Using filtered gene expression profiles, we identified statistically significant differentially expressed genes of CD4-GZMA cells using R script provided by the guideline (12). The cut-off criteria were false discovery rate (FDR) of <0.05 and |log2 fold change| > 1.0.

### Construction and Validation of an Immune Risk Score Model

We applied the univariable Cox proportional hazards regression to calculate the hazard proportions for CD4-GZMA cell specific DEGs (FDR <0.05, |log2 fold change| > 1.0). Among DEGs with significance at *p* < 0.05, we used LASSO Cox regression to select the most useful prognostic genes. To improve the robustness of the LASSO Cox regression model, we repeated the LASSO Cox regression fitting process for 10-fold cross-validation evaluations 1,000 times. Genes with non-zero coefficient estimates in at least 900 of these 1,000 evaluations were chosen for the final model. Based on the average coefficient of each gene, a formula for the immune risk score model was established to predict patient survival:

$$\begin{array}{l} \text{Immune risk score}\\ =\sum (Cox\;coefficient\;of\;gene\;Xi\;*\;scale\;expression\;value\;of\;gene\;Xi) \end{array}$$

Coefficients were the following: *FNDC4*: 0.004549632; *RNF186*: 0.10682142; *PKIB*: 0.006859035; *MIR3609*: −0.215215851; *PLEKHA4*: 0.004534235; *ANKRD24*: −0.05629549; *CEACAM19*: −0.009974964; *DIO3OS*: −0.050176196; *UBASH3A*: −0.045030993; *KCNE5*: 0.017597103; *PCAT6*: 0.006024639; and *CCDC184*: −0.503357872; *XCR1*: −0.059899642.

Using R package “survminer” to evaluate the optimal cut-off values of the risk score, the Kaplan-Meier analysis were conducted in datasets from TCGA and ICGC for validation of the model.

### Construction and Validation of the Nomogram Model

A nomogram was established to visualize the prognostic value of the immune risk score using R package “rms.” The calibration curves were plotted to assess the predicted probabilities in comparison with the best predictive line. To determine the predictive accuracy of the nomogram, we calculated the concordance index(C-index) using R package “survcomp.” In addition, we applied time-dependent receiver operating characteristic (ROC) curve and Decision Curve Analysis (DCA) to evaluate the performance of the nomogram in predicting overall survival (OS) in different years with R package “ROCR” and “rmda.”

### Statistical Analyses

The Wilcoxon test was used to estimate the statistical significance for 11 cell subsets' distribution between tumor and normal groups. The optimal cut-off values based on the association between overall survival and cell fraction or risk score in each dataset were evaluated by R package “survminer.” Survival curves were generated by the Kaplan-Meier method and compared by means of the log rank test using “survival” package. We used the univariable Cox proportional hazards regression model to calculate a hazard ratio for univariable analysis. Using the “glmnet” package, the LASSO Cox regression algorithm with internal 10-fold cross-validation was applied to select the most useful prognostic genes. We used time-dependent ROC curve to depict the sensitivity and specificity of the survival prediction based on the immune risk score. The quantification of the area under the ROC curve were calculated using the “ROCR” package. All statistical analyses were conducted using R software (version 3.6.1). The R codes involved in this study could be downloaded from the link https://github.com/szlilixing/Transcriptome-analysis. A two-tailed *P* < 0.05 were considered statistically significant.

## Results

### Create a Signature Matrix of 11 Immune Cell Subsets From scRNA-Seq Data

We acquired 4,070 cell samples clustered from 11 immune cell subsets isolated from peripheral blood, tumor, and adjacent normal tissues from hepatocellular carcinoma patients (Table 1). The cell clusters included C1_CD8-LEF1, C2_CD8-CX3CR1, C3_CD8-SLC4A10, C4_CD8-LAYN, C5_CD8-GZMK, C6_CD4-CCR7, C7_CD4-FOXP3, C8_CD4-CTLA4, C9_CD4-GZMA, C10_CD4-CXCL13, and C11_CD4-GNLY (7). Based on the CIBERSORTx algorithm, a signature matrix including 2,527 genes of 11 cell clusters was created (Supplementary Figure 1A, Supplementary Table 1).

**Table 1:** T cells isolated from peripheral blood, tumor, and adjacent normal tissues from hepatocellular carcinoma patients in GSE98638 were finally analyzed in this study.

**Category**	**Cell counts**	**Percentage (%)**
C01_CD8-LEF1	161	3.96
C02_CD8-CX3CR1	288	7.08
C03_CD8-SLC4A10	363	8.92
C04_CD8-LAYN	300	7.37
C05_CD8-GZMK	467	11.47
C06_CD4-CCR7	646	15.87
C07_CD4-FOXP3	261	6.41
C08_CD4-CTLA4	582	14.30
C09_CD4-GZMA	689	16.93
C10_CD4-CXCL13	146	3.59
C11_CD4-GNLY	167	4.10
Total	4,070	100

### Identification of Immune Infiltration of Bulk Samples Based on Signature Matrix

**Figure 2:**
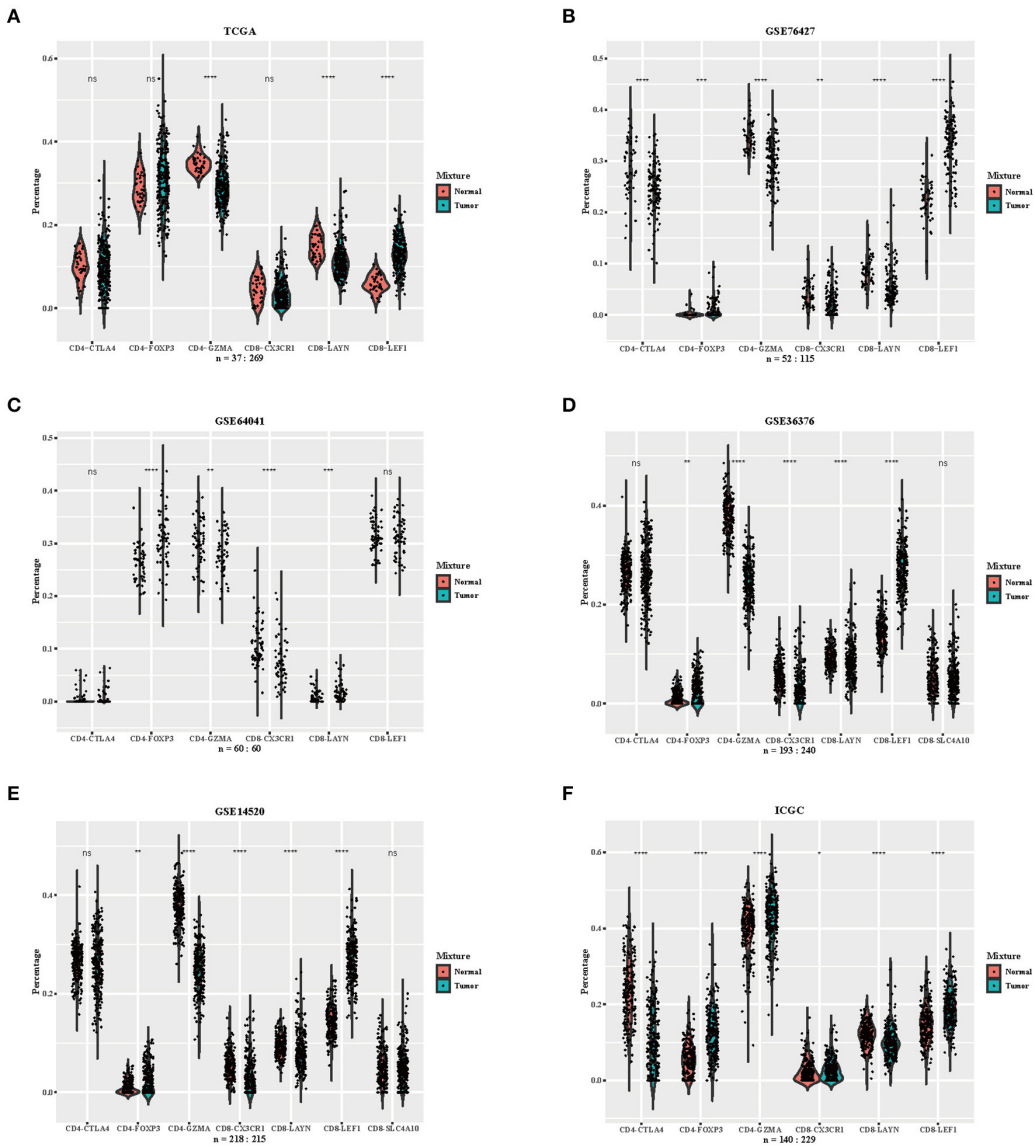
Distribution of inferred immune cell subsets in various datasets. **(A–F)** Violin plots depicting the different distributions of several immune cell subsets between tumor and normal samples from datasets TCGA, GSE76427, GSE64041, GSE36376, GSE14520, and ICGC (Immune cell subsets with low proportions were excluded, depicted *p*-values are from Wilcoxon test). ns, not significant; *p < 0.05; **p < 0.01; ***p < 0.001; ****p < 0.0001.

We downloaded RNA-seq profiles or gene expression profiles of HCC patients from TCGA, GSE76427, GSE64041, GSE36376, GSE14520, and ICGC. Using the CIBERSORTx algorithm, we calculated the relative proportion of immune subsets between tumor and normal samples (Figure 2). CD4-GZMA cells and CD8-LAYN cells were found to have higher infiltration in normal tissue while CD8-LEF1 cells showed higher fraction in tumor tissue (Figure 2). To some extent, the fraction of CD4-FOXP3 cells were also lower in normal tissue from most datasets. The distribution of CD8-CX3CR1 cells showed a slightly decrease in tumor sites. The CD4-CTLA4 cells had no significant differences between tumor and normal samples. The rest of the cell subsets had low levels in both tumor and normal samples (data not shown).

### Correlation of Proportion of Immune Subsets With Overall Survival

**Figure 3:**
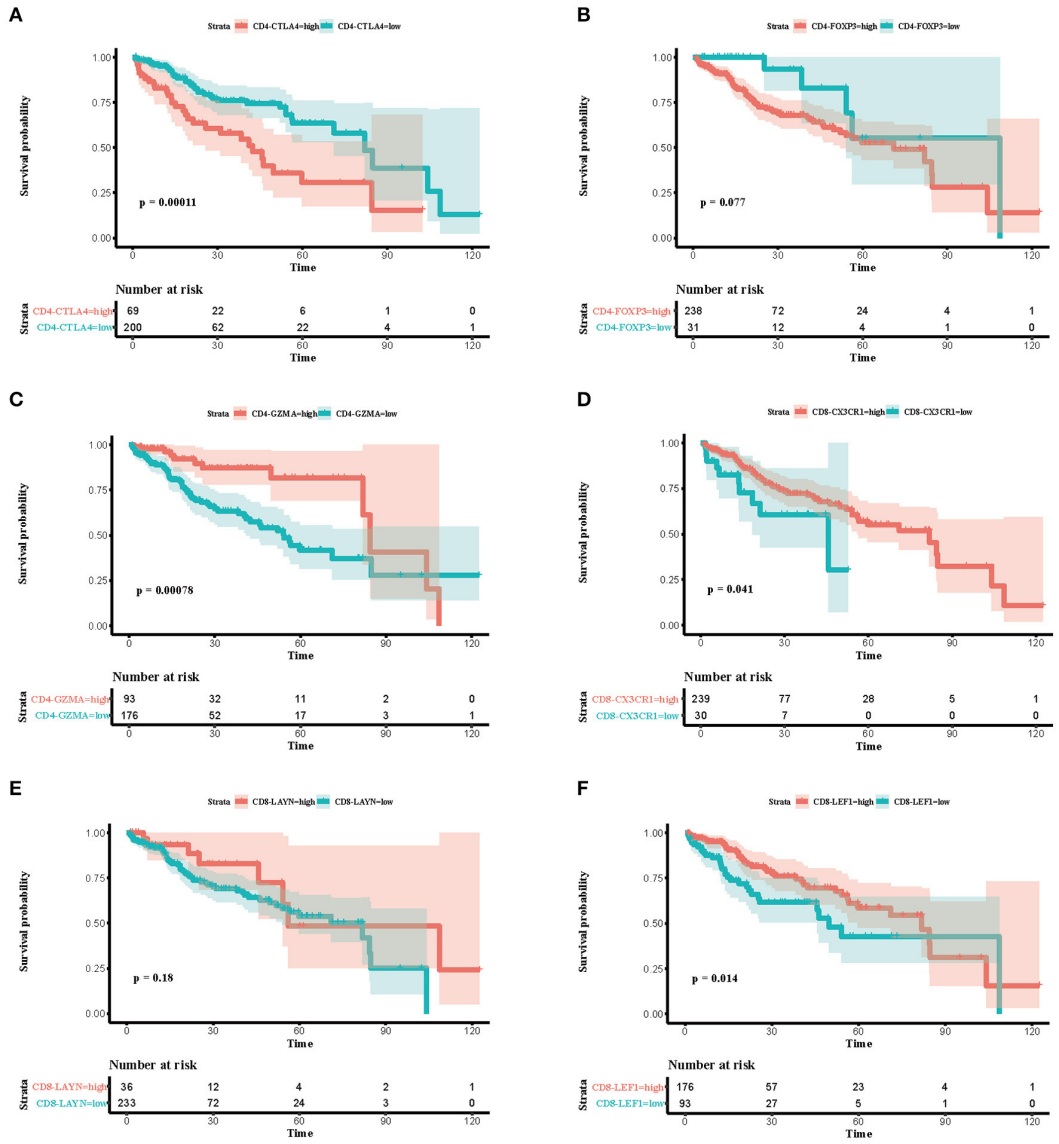
Associations between survival and six immune cell subsets in the TCGA cohort. **(A–F)** Survival plots of immune cell subsets in the TCGA cohort. Time was calculated by month. Depicted *p*-values are from log-rank tests.

To explore the prognostic value of immune subsets, we first evaluated the association between overall survival and cell fraction in the TCGA cohort (Table 2) using R package “survminer” (Figures 3A–F). A high proportion of CD4-CTLA4 cells and a low proportion of CD4-GZMA cells were significantly associated with poor overall survival in log-rank test (Figures 3A,C). Besides, CD8-CX3CR1 and CD8-LEF1 cells were also associated with prognosis in the TCGA cohort (Figures 3D,F). However, we only found lower infiltration of CD4-GZMA cells associated with poor prognosis in two other datasets (Table 2) (Supplementary Figures 2A,B).

**Table 2:** Clinical characteristics of the patients in this study.

***N***	**TCGA LIHC (*N* = 269) (%)**	**ICGC LIRI (*N* = 229) (%)**	**GSE14520 (*N* = 213) (%)**
Age, median (range)	60 (16–82)	67 (31–89)	59 (21–77)
**Gender**			
Female	85 (31.60%)	61 (26.64%)	30 (14.08%)
Male	184 (68.40%)	168 (73.36%)	183 (85.92%)
**TNM stage**			
I	153 (56.88%)	36 (15.72%)	90 (42.25%)
II	63 (23.42%)	105 (45.85%)	75 (35.21%)
III	50 (18.59%)	69 (30.13%)	48 (22.54%)
IV	3 (1.11%)	19 (8.30%)	
**Histological type**		Not report	
Hepatocellular carcinoma	263 (97.78%)		213 (100%)
Fibrolamellar carcinoma	2 (0.74%)		
Hepatocholangio carcinoma	4 (1.48%)		

*TCGA, The Cancer Genome Atlas; ICGC, International Cancer Genome Consortium; LIHC, Liver hepatocellular carcinoma; LIRI-JP, Liver Cancer-RIKEN, JP*.

### Differentially Expressed Genes Analysis and Functional Enrichment Analysis

**Figure 4:**
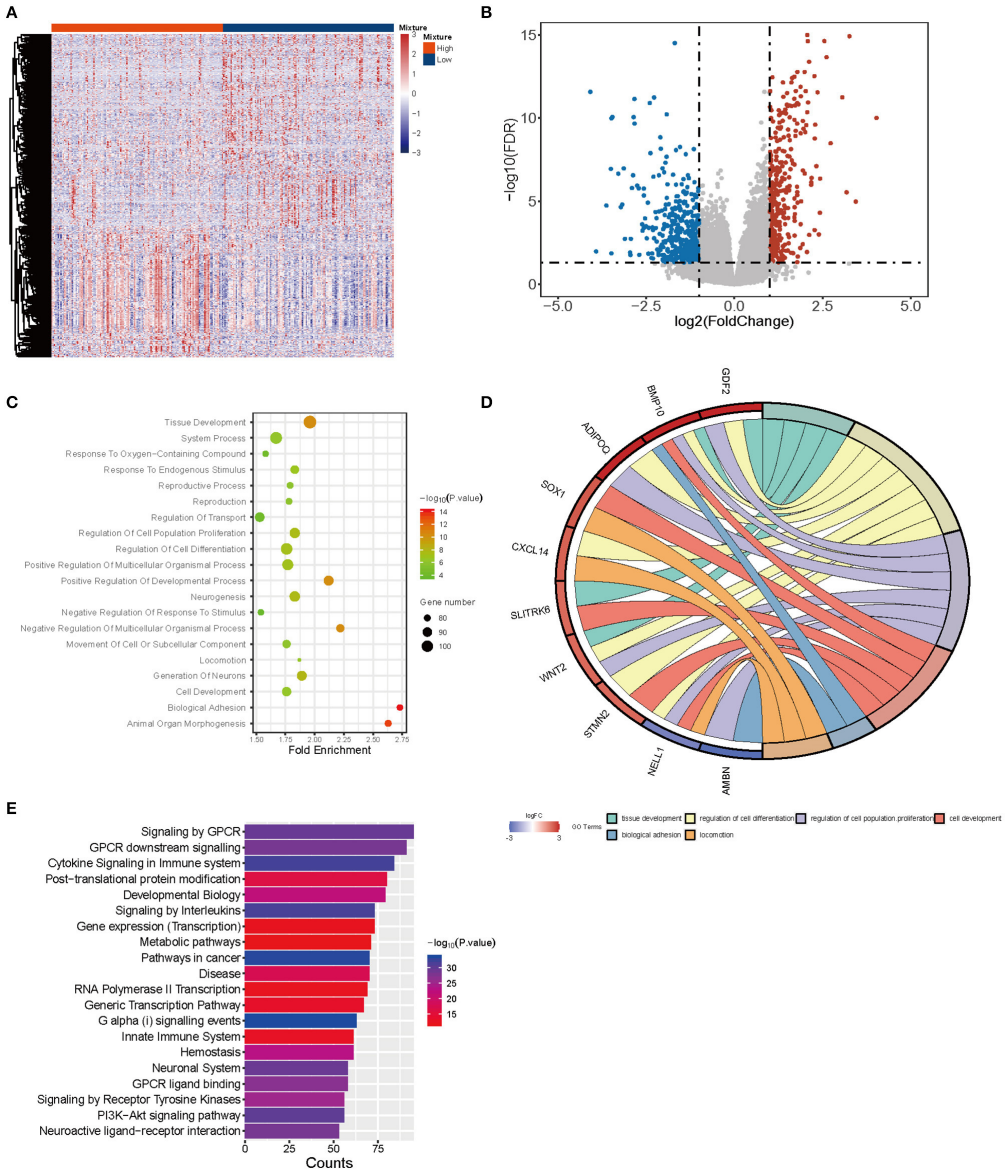
The differentially expressed genes between CD4-GZMA high and low groups defined by median proportion. **(A)** The heatmap of differentially expressed genes between CD4-GZMA high and low groups. **(B)** The volcano Plot of differentially expressed genes between CD4-GZMA high and low groups. **(C)** GO analysis of differentially expressed genes. **(D)** The chord diagram of GO analysis. **(E)** Pathway Enrichment of differentially expressed genes.

Considering different proportions in tumor and normal tissues, CD4-GZMA cells may play an important role in HCC patients' prognosis. Thus, we analyzed the differentially expressed genes between high and low groups according to the proportion of CD4-GZMA cells (the cut-off value was the media of fraction of CD4-GZMA cells). We set the cut-off criteria as |log2 fold change| > 1.0 and adj.p <0.05 and identified 755 differentially expressed genes (DEGs) (Figures 4A,B). Functional enrichment clustering of these genes showed strong association with the tumor microenvironment and immune response (Figures 4C–E).

### CIBERSORTx Group-Mode Analysis

**Figure 5:**
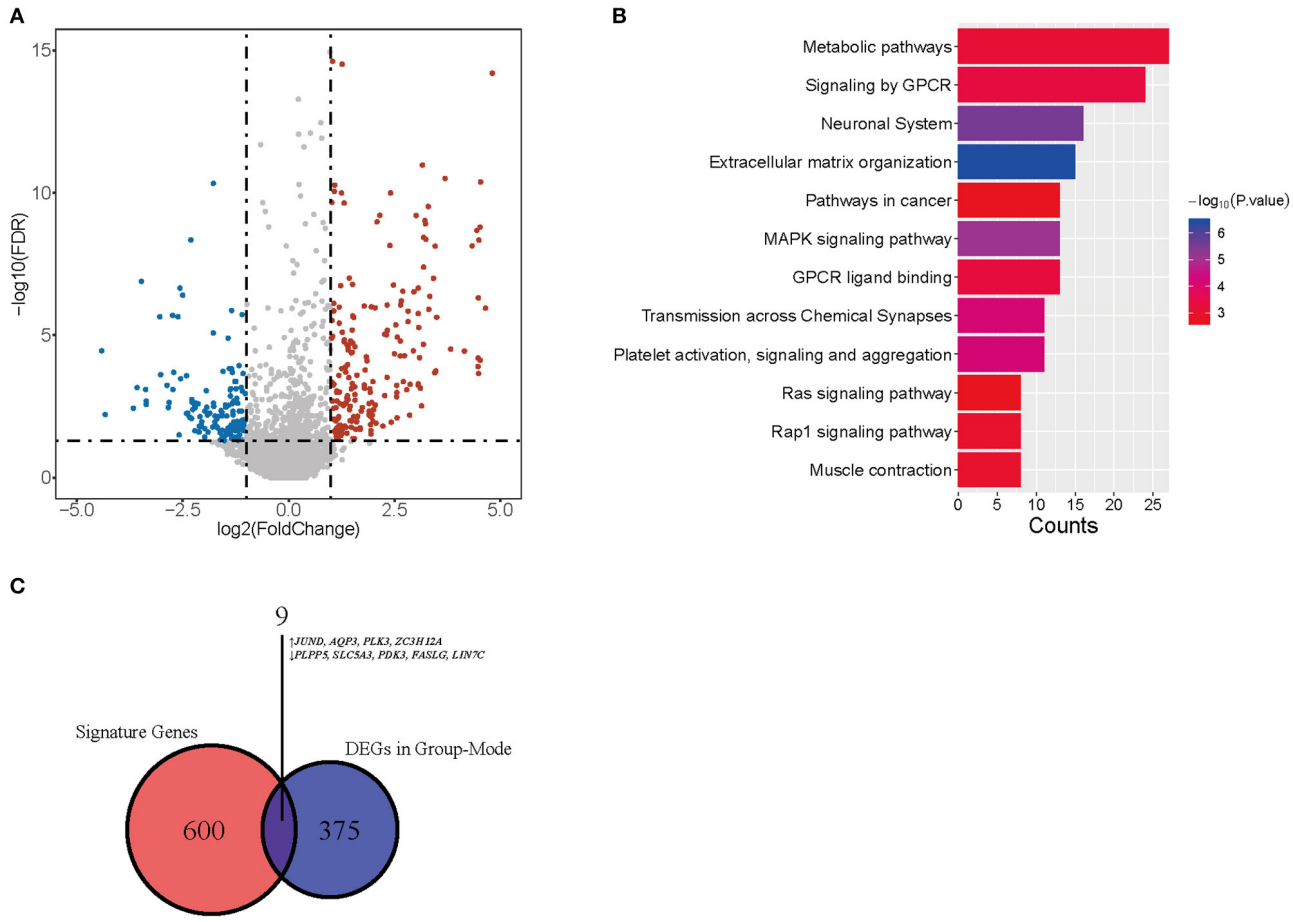
CD4-GMZA-specific differentially expressed genes. **(A)** The volcano plot of CD4-GMZA cell-specific differentially expressed genes between tumor and normal tissues by group-mode of CIBERSORTx. **(B)** Pathway Enrichment of differentially expressed genes. **(C)** The Venn diagram between CD4-GZMA cell specific differentially expressed genes and signature genes of CD4-GZMA cell subset. ↑, Up-regulated genes; ↓, Down-regulated genes.

We performed CIBERSORTx group-mode analysis to impute CD4-GZMA cell specific gene expression according to the guidelines (12). Setting cut-off criteria as false discovery rate (FDR) of <0.05 and |log2 fold change| > 1.0, we identified 384 differentially expressed genes (DEGs) (Figure 5A). Pathway analysis showed associations with cancer, metabolism, and immunity (Figure 5B). In addition, the Venn diagram showed nine genes appearing in both CD4-GZMA cell specific differentially expressed genes and signature genes of the CD4-GZMA cell subset (Figure 5C).

### Construction and Validation of Immune Risk Score Mode

To further mine the potential prognostic value of CD4-GZMA cell specific DEGs, univariable cox survival analysis were first applied to find out their prognostic role, and 19 genes were finally observed associated with HCC patients' prognosis (Table 3).

**Table 3:** Univariable cox analysis of CD4-GZMA cell-specific different expressed genes detected in group mode with CIBERSORTx.

**Gene**	**HR**	**95% CI**	**wald.test**	**p.value**
*FNDC4*	1.3	1.1–1.6	8.2	0.0041
*RNF186*	1.4	1.1–1.9	6.5	0.011
*PKIB*	1.2	1–1.4	5.9	0.015
*MIR3609*	0.63	0.43–0.92	5.8	0.016
*PLEKHA4*	1.3	1–1.6	5.7	0.017
*LINC01370*	1.1	1–1.2	5.6	0.018
*ANKRD24*	0.68	0.49–0.94	5.4	0.02
*CEACAM19*	0.73	0.56–0.95	5.3	0.021
*DIO3OS*	0.73	0.56–0.95	5.3	0.021
*GJA4*	0.77	0.61–0.97	4.8	0.029
*NLRC3*	0.6	0.37–0.95	4.7	0.03
*UBASH3A*	0.65	0.45–0.96	4.7	0.03
*KCNE5*	1.3	1–1.6	4.6	0.032
*PCAT6*	1.3	1–1.5	4.6	0.032
*TAPT1*	0.68	0.48–0.97	4.6	0.032
*TMEM88*	0.77	0.59–0.99	4.2	0.041
*CCDC184*	0.48	0.24–0.97	4.1	0.042
*XCR1*	0.52	0.28–0.98	4.1	0.043
*CASQ2*	0.75	0.57–1	4	0.047

**Figure 6:**
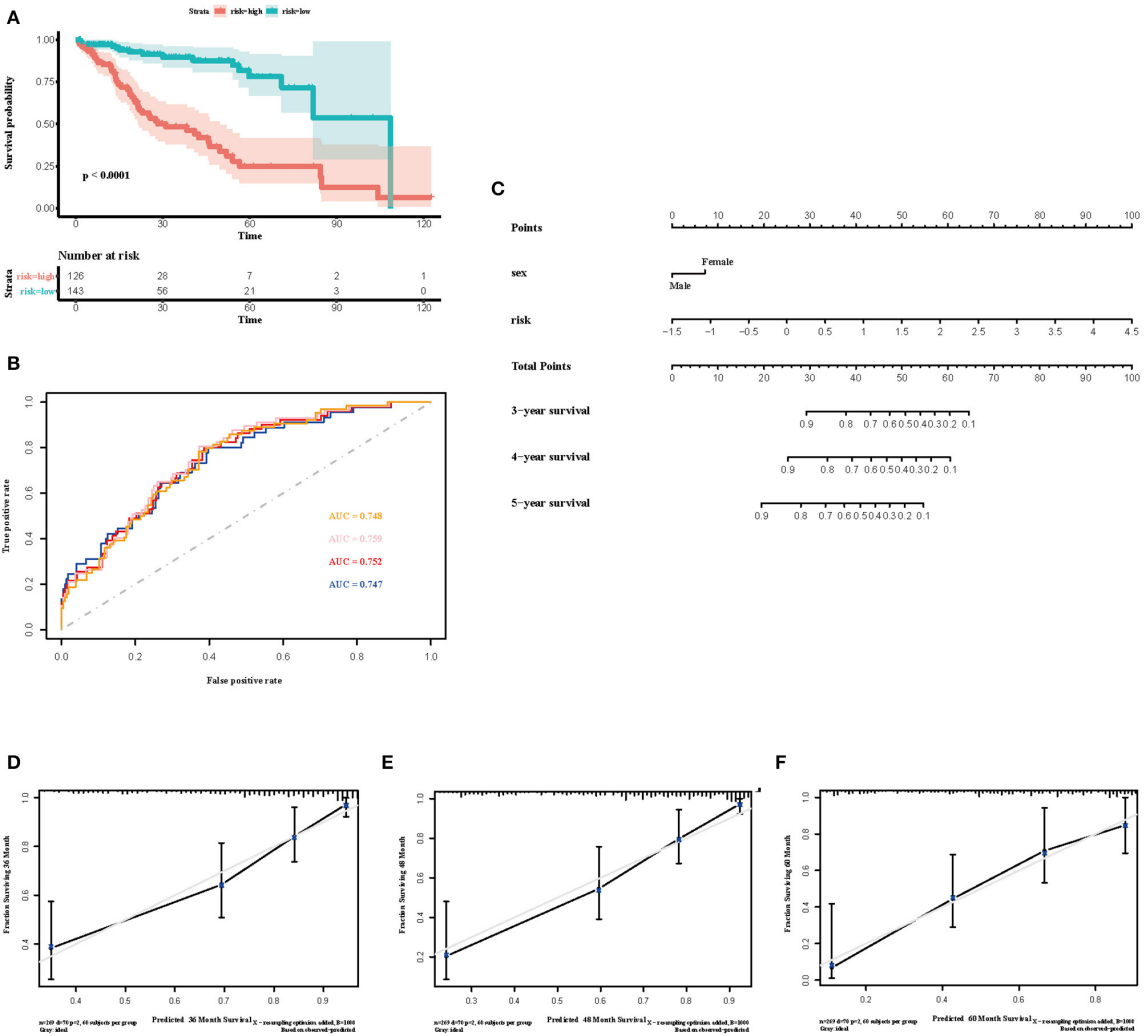
Construction of the immune risk score model. **(A)** Kaplan-Meier curve for overall survival by risk score group in TCGA cohort. Time was calculated by month. **(B)** Time-dependent ROC curve in the TCGA cohort. The area under the ROC curve were 0.748, 0.759, 0.752, and 0.747 for the risk score at 2, 3, 4, and 5 years, respectively. **(C)** The nomogram to visualize the prognostic value of the immune risk score in the TCGA cohort. **(D–F)** The calibration curves to assess the predicted probabilities in comparison with the best predictive line at 3, 4, and 5 years, respectively.

**Figure 7:**
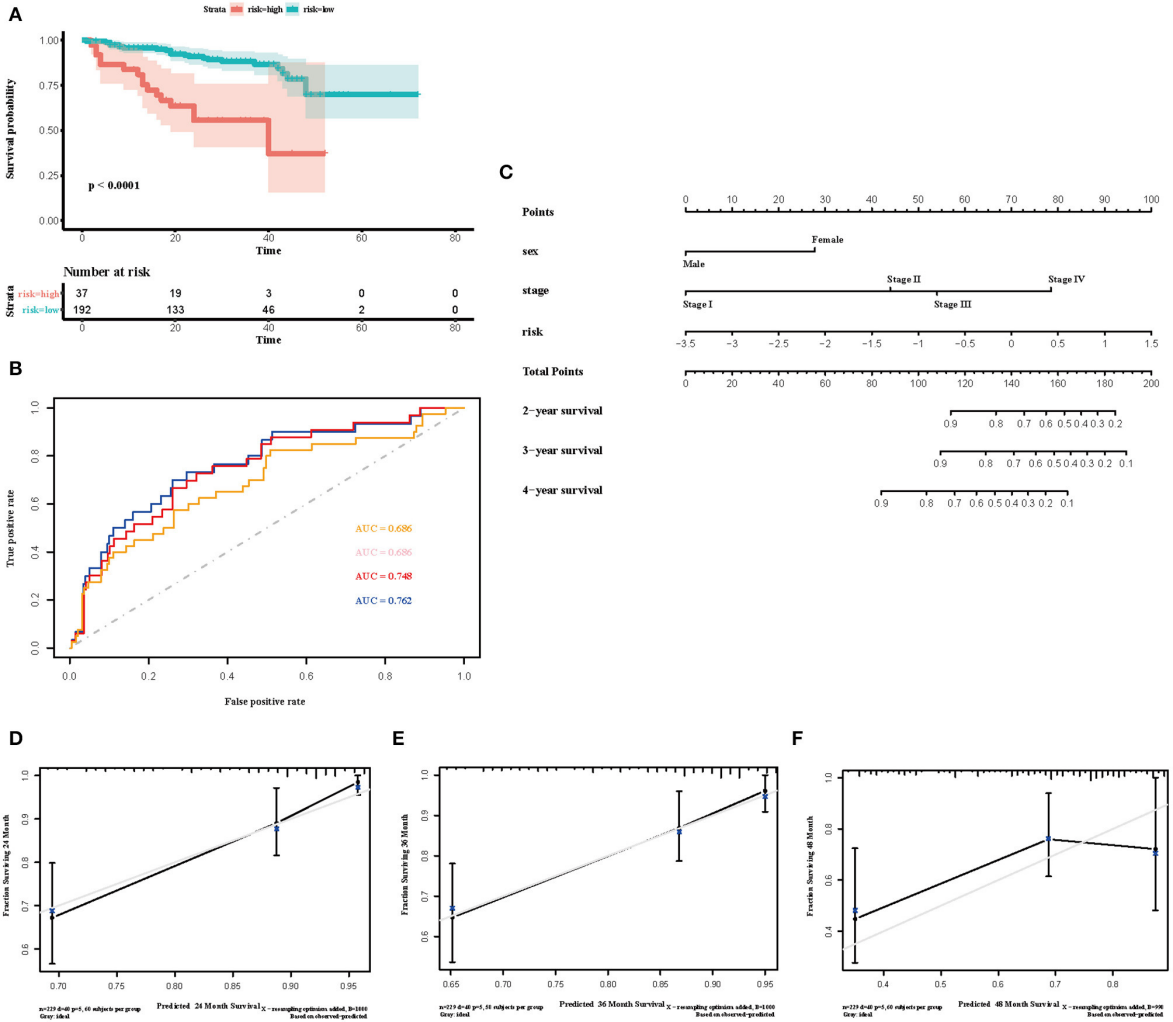
Validation of the immune risk score model in the ICGC cohort. **(A)** Kaplan-Meier curve for overall survival by risk score group in the ICGC cohort. Time was calculated by month. **(B)** Time-dependent ROC curve in the ICGC cohort. The area under the ROC curve were 0.686, 0.686, 0.748, and 0.762 for the risk score at 2, 3, 4, and 5 years, respectively. **(C)** The nomogram to visualize the prognostic value of the immune risk score in the ICGC cohort. **(D–F)** The calibration curves to assess the predicted probabilities in comparison with the best predictive line at 2, 3, and 4 years, respectively.

To select the most useful prognostic genes, LASSO Cox regression analysis was used to build an immune risk score model in the TCGA cohort (Figure 6). We repeated the LASSO Cox regression fitting process for 10-fold cross-validation evaluations 1,000 times to improve the accuracy of the model (Supplementary Figures 3A,B). Thirteen genes with non-zero coefficient estimates in at least 900 of these 1,000 evaluations were finally selected for the LASSO model. The formula for the immune risk score can be found in materials and methods. The Kaplan-Meier analysis of the TCGA cohort showed strong association between overall survival and the risk score. We investigated the prognostic accuracy of the model in the TCGA cohort using time-dependent ROC analysis at the time points 2, 3, 4, and 5 years (Figure 6B). Moreover, we constructed a nomogram to visualize the prognostic value of the immune risk score (Figure 6C). The concordance index(C-index) was 0.775. The calibration curves showed well-predicted probabilities compared with the best predictive line (Figures 6D–F). In addition, we validated our model in the dependent dataset from ICGC (Figure 7). After building a nomogram, the concordance index(C-index) was calculated as 0.787 (Figure 7C). Moreover, we evaluated the performance of the nomogram in predicting OS in different years using the time-dependent ROC curve and Decision Curve Analysis (DCA) (Supplementary Figures 4, 5).

## Discussion

Despite substantial progress having been made in treating HCC, the implementation of effective precision medicine remains challenging (3, 18). Identification of the characteristics of the tumor microenvironment, especially immune context and robust predictive biomarkers, may ultimately improve the clinical management of HCC (5, 19). Advances in scRNA-seq have allowed us to explore detailed compositions in tumors at high resolutions. A series of recent studies using the scRNA-seq technique have been conducted to disclose the mystery of HCC (7, 8, 20–23). In the current work, we aimed to explore the tumor-infiltration and prognostic value of new cell subsets identified by a recent HCC scRNA-seq study, using the state-of-the-art deconvolution algorithm CIBERSORTx (7, 11). The scRNA-seq study has already identified 11 TILs of HCC, including many exhausted T cells (7). The signature matrix of 11 immune cell subsets created with CIBERSORTx involved more genes and partly overlapped with signature genes of the scRNA-seq data. According to the signature matrix, CD8-LAYN cell and CD4-CTLA4 cell were characterized by *PDCD1* (programmed cell death 1) and *CTLA4* (cytotoxic T-lymphocyte associated protein 4), indicating their exhausted function (24). However, we also observed high level expression of effective functional genes, such as *GZMA* (granzyme A) and *GZMB* (granzyme B), in CD8-LAYN cell. This subset might be involved in the regulation of tumor immunity through a dual functional mechanism. The CD4-GZMA cell type might play a positive role in HCC immunity on account of its high expression of effective immune genes, especially *GZMA*.

Due to a lack of robust effector functions, exhausted T cells express multiple inhibitory receptors with an altered transcriptional programme (25). An abundance of exhausted T cells results in an invalid control of tumors, leading to poor prognosis (26–29). Yet, after deconvolution of the TCGA HCC RNA-seq data with CIBERSORTx, the proportion distribution of 11 cell types (mainly six cell types) in tumor and normal tissues showed a different trend. Firstly, among relatively high proportion CD8^+^ cell subsets, CD8-LAYN cells' infiltration was higher in normal tissues than tumorous ones without association with prognosis. We inferred that CD8-LAYN cell might immigrate from normal to tumor sites and lose its function gradually (25). The complex regulatory mechanism behind this process remained unclear. Secondly, a high fraction of the CD4-CTLA4 subset was statistically significantly associated with poor prognosis in two datasets, though there was no difference in their proportion between tumor and normal tissues. Considering the high expression of exhausted functional genes, including *PDCD1* and *CTLA4*, the CD4-CTLA4 cell subset might also be a potential target for immunotherapies. Thirdly, the CD4-GZMA cell subset showed great prognostic value combined with their different abundance between tumor and normal tissues. CD4^+^ T cells have been proven to participate in anti-tumor immunity and improving prognosis (30, 31). The CD4-GZMA cell subset is similar to Th1 cells (7). We assumed the CD4-GZMA cell type might be a key point in anti-tumor immune responses in HCC.

To further explore the role of the CD4-GZMA cell subset in HCC development, we analyzed the differentially expressed genes (DEGs) between high and low CD4-GZMA cell infiltrated groups and further performed GO and Pathway analysis. Among these DEGs, 323 genes were up-regulated, and 432 genes were down-regulated. Biology process analysis showed aberrations of growth, differentiation, and organization of cell populations happened in the high CD4-GZMA cell infiltrated group. Among the core DEGs of GO function, *BMP10* (bone morphogenetic protein 10) and *KCNK2* (potassium two pore domain channel subfamily K member 2) were proven to be associated with progression and prognosis in HCC (32, 33). *OR7C1* (olfactory receptor family 7 subfamily C member 1) represented a novel marker of colon cancer-infiltrating cells (CICs) (34). Considering its higher expression in the CD4-GZMA highly infiltrated group, it may help promote CTL-like immune responses against tumors. In addition, up-regulated *BMP9* (bone morphogenetic protein 9, also known as *GDF2*, growth differentiation factor 2) were involved in cell migration and epithelial to mesenchymal transition (EMT) in HCC (35). Pathway analyses were highly associated with immune responses, including cytokine signaling in the immune system, signaling by Interleukins, and the innate immune system. The core up-regulated genes were *FGF23* (fibroblast growth factor 23), *MUC5AC* (mucin 5AC), *FCN3* (ficolin 3), and *C7* (complement C7). Ficolin 3 expressed typically in the lung and liver, playing a vital role in innate immunity. The mutation or deficiency of *FCN3* may cause immune disorders like SLE (Systemic Lupus Erythematosus) (36–38). In addition, GPCR-related pathways were enriched, possibly serving as important regulators in HCC development (39–41). Using group-mode of CIBERSORTx, we estimated CD4-GZMA cell specific gene expression between tumor and normal samples (11, 12). The CD4-GZMA cell specific genes were enriched in pathways associated with tumor metabolism and malignant progression. Moreover, there were nine genes appearing in both CD4-GZMA cell specific differentially expressed genes and signature genes of the CD4-GZMA cell subset. We inferred that they may exert great effectiveness in HCC progression and tumor immunity. Previous studies have reported that *JUND* (JunD proto-oncogene), *AQP3* [aquaporin 3 (Gill blood group)], and *PLK3* (polo like kinase 3) promote hepatocarcinogenesis and metastasis (42–44). *PLPP5* (phospholipid phosphatase 5, or *HTPAP*) was defined as a metastatic suppressor of HCC, which was down-regulated in tumor samples (45). *ZC3H12A* (zinc finger CCCH-type containing 12A) was involved in cellular inflammatory response and immune homeostasis and it negatively regulates Interleukin-17-Mediated Signaling (46). Mutations of *ZC3H12A* had recently been verified to be associated with ulcerative colitis recently (47, 48). Besides, *SLC5A3* (solute carrier family 5 member 3) was proved to be promote inflammatory responses in inclusion body myositis (IBM) (49). Their up-regulation might further reduce the anti-tumor immune response. Down-regulation of *LIN7C* (lin-7 homolog C) was found to be related to oral squamous cell carcinoma (OSCC) metastasis in an early research (50). In addition, *PDK3* (pyruvate dehydrogenase kinase 3) had been reported to regulate tumor cell differentiation and cell fate. Yet, it remained unclear how they work in HCC progression. *FASLG* (Fas ligand), a core gene involved in lymphocyte apoptosis and T-cell development, was down-regulated, which meant partial loss of self-regulation in tumor immunity.

In recent years, various immunoscore models, based on TILs' proportion, ratio of immune cells, and expression of prognostic genes, have been developed and verified to assess prognosis (51–53). Though current studies showed relatively good probability of prognostic assessment in part, they focus on the overall distribution of immune cells and total gene expression difference between samples. In this study, we estimated CD4-GZMA cell specific genes with CIBERSORTx in the TCGA HCC dataset and evaluated their prognostic value using univariable cox survival analysis. We further applied LASSO cox regression to construct our immune risk model for higher accuracy (54). Time-dependent ROC analysis and validation independent of the dataset ICGC demonstrated the reliability of our model. We also established a nomogram model to visualize the prognostic value of the immune risk score. Thirteen genes were incorporated in our model. Among these genes, previous studies had reported that *FNDC4* (fibronectin type III domain containing 4), *RNF186* (ring finger protein 186), and *UBASH3A* (ubiquitin associated and SH3 domain containing A) were associated with inflammatory bowel disease (55–57). *PKIB*(cAMP-dependent protein kinase inhibitor beta) was reported as a key regulator of the *PI3K/Akt* pathway involved in tumor aggressiveness in NSCLC (non-small cell lung cancer) and prostate cancer (58, 59). *MIR3609* (microRNA 3609) had been proven to improve the immune response in breast cancer by blocking the programmed death-ligand 1 immune checkpoint (60). *PLEKHA4* (pleckstrin homology domain containing A4) might be involved in the progression of a tumor through the *Wnt* pathway (61). *CEACAM19* (CEA cell adhesion molecule 19), *DIO3OS* (DIO3 opposite strand upstream RNA), and *PCAT6* (prostate cancer associated transcript 6) were verified as associated with the prognosis of different cancers, including gastric cancer, breast cancer, liver cancer, and lung cancer (62–64). *KCNE5* (potassium voltage-gated channel subfamily E regulatory subunit 5) was more closely related to heart disease (65). How they play a role in HCC progression requires further study. *XCR1* (X-C motif chemokine receptor 1) and *XCL1* (X-C motif chemokine ligand 1) had been reported to enhance proliferation of antigen-specific CD8^+^ T cells and their anti-tumor immunity (66, 67). *ANKRD24* (ankyrin repeat domain 24) and *CCDC184* (coiled-coil domain containing 184) lack research information. CD4-GZMA cell specific genes with a prognostic value in our model might shed new light on the progression and prognosis of HCC and help obtain better outcomes in clinical combination therapy.

There are some limitations to our study. First, the datasets we analyzed are based on the public database. Thus, the information is insufficient, especially regarding the lack of clinical details to improve prognostic accuracy. We also lack ground truth data to further validate our results with CIBERSORTx. The exact regulatory mechanisms of these new TILs remain unclear. Whether immune characteristics we found in HCC are indictive of suitable immunotherapies needs further study. Finally, we mainly re-analyzed CD4^+^ and CD8^+^ T cell clusters identified by a scRNA-seq dataset and the risk score is limited in scope to reflect a comprehensive immune status in HCC tissue.

In sum, using a machine learning method CIBERSORTx, we evaluated the infiltration of new immune subsets identified by a scRNA-seq date in TCGA HCC samples. Effective CD8^+^ T cell subsets generally had a low proportion in tumor sites, leading to an ineffective immune response. Yet, the new subset CD4-GZMA cell may exert significant levels of anti-tumor immunity. Considering their Th1-like characteristics, there may be tissue-specific neoantigen in HCC still to discover. Thus, the CD4-GZMA cell type could be a potential target of immunotherapies. Further study is needed to explore the exact mechanism during anti-tumor immunity and find out the neoantigens in HCC. Moreover, the outstanding CIBERSORTx algorithm provides us opportunities regarding cell-specific gene expression. We located several key genes associated with prognosis and built a useful model for predicting HCC patient's outcome.

## Data Availability Statement

All datasets generated for this study are included in the article/Supplementary Material.

## Author Contributions

TX and SQ were involved in the design of the work, analysis of data, and approval of the final version to be published. JM and QZ contributed to statistical analysis of the data and drafting the manuscript. ML, HW, MW, DZ, and TW contributed to acquisition and analysis of data. LL and LS were responsible for drafting the manuscript, analysis, and interpretation of the data. All authors read and approved the final manuscript.

## Conflict of Interest

The authors declare that the research was conducted in the absence of any commercial or financial relationships that could be construed as a potential conflict of interest.

## Supplementary Material

Supplementary Figure 1 Signature gene matrix of inferred immune cell subsets by study. **(A)** Heatmap showing a custom signature matrix created from scRNA-seq data (GSE98638) with CIBERSORTx.

Supplementary Figure 2 Association between survival and four immune cell subsets in ICGC and GSE14520 cohorts. **(A,B)** Survival plots of immune cell subsets (CD4-GZMA, CD4-CTLA4, CD8-CX3CR1, CD8-LEF1) in ICGC **(A)** and GSE14520 **(B)** cohorts. Time was calculated by month. Depicted *p*-values are from log-rank tests.

Supplementary Figure 3 The frequency and coefficients of representative genes during 1,000 iterations of LASSO Cox regression. **(A)** Frequency of representative genes investigated in 1,000 iterations of LASSO Cox regression. **(B)** Box plot for mean and confidence intervals of coefficients.

Supplementary Figure 4 The evaluation of the performance of the nomogram from TCGA cohort in predicting OS in different years. **(A)** Time-dependent ROC curve of the nomogram. The area under the ROC curve were 0.764, 0.786, and 0.775 for the risk score at 3, 4, and 5 years, respectively. **(B–D)** The Decision Curve Analysis (DCA) of the nomogram.

Supplementary Figure 5 The evaluation of the performance of nomogram from ICGC cohort in predicting OS in different years. **(A)** Time-dependent ROC curve of the nomogram. The area under the ROC curve were 0.764, 0.786, and 0.775 for the risk score at 2, 3, and 4 years, respectively. **(B–D)** The Decision Curve Analysis (DCA) of the nomogram.

Supplementary Table 1 Signature matrix with CIBERSORTx based on a scRNA-seq study (GSE98638).

Supplementary Table 2 Clinical information of TCGA LIHC samples.
