# Supplementary figures and images for: Evaluating Distribution and Prognostic Value of New Tumor-Infiltrating Lymphocytes in HCC Based on a scRNA-Seq Study With CIBERSORTx
Source: Front Med (Lausanne). 2020 Sep 17;7:451. doi: 10.3389/fmed.2020.00451 (PMC7527443; doi:10.3389/fmed.2020.00451)

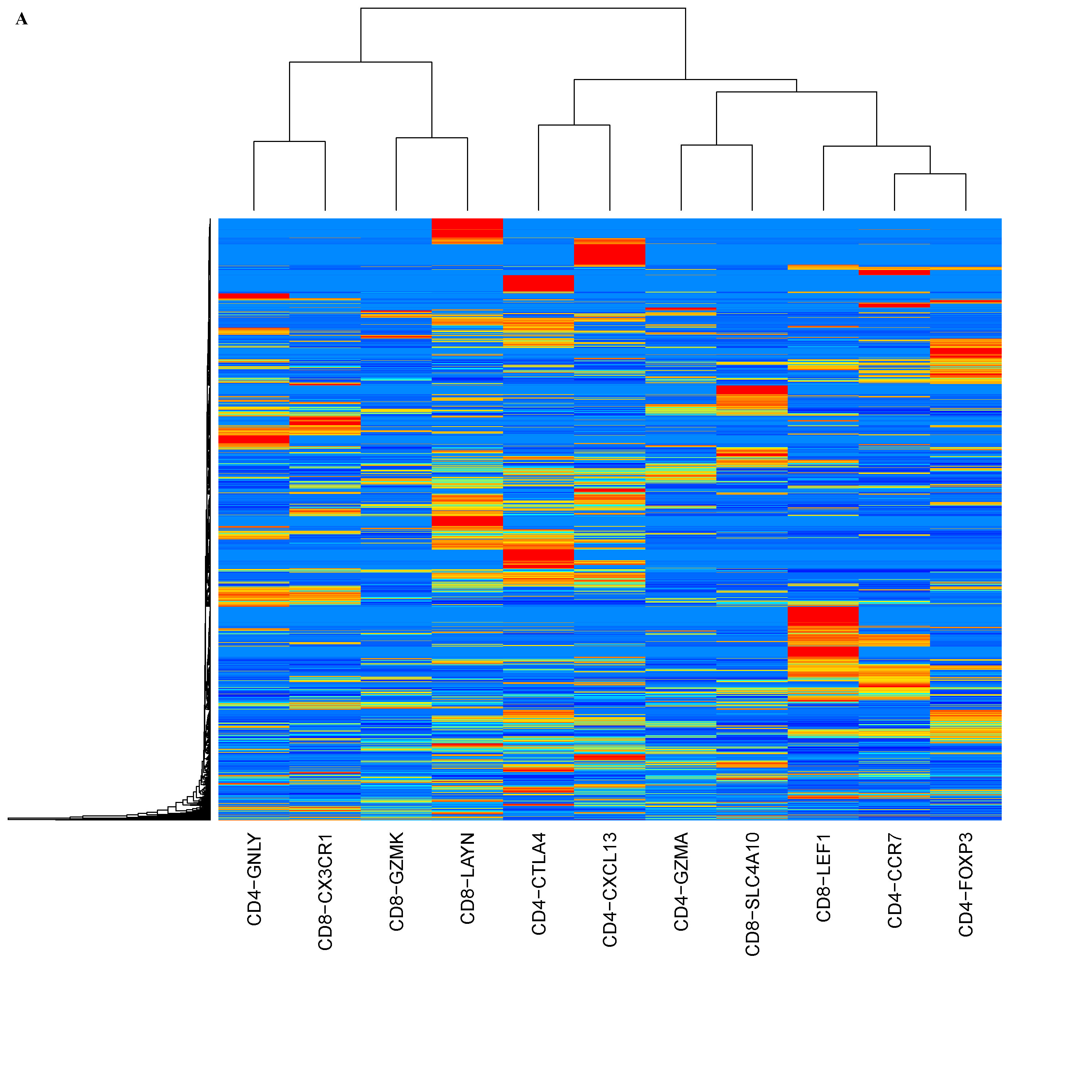

Supplement: Supplementary Figure 1 — Signature gene matrix of inferred immune cell subsets by study. (A) Heatmap showing a custom signature matrix created from scRNA-seq data (GSE98638) with CIBERSORTx. [file Image_1.JPEG]

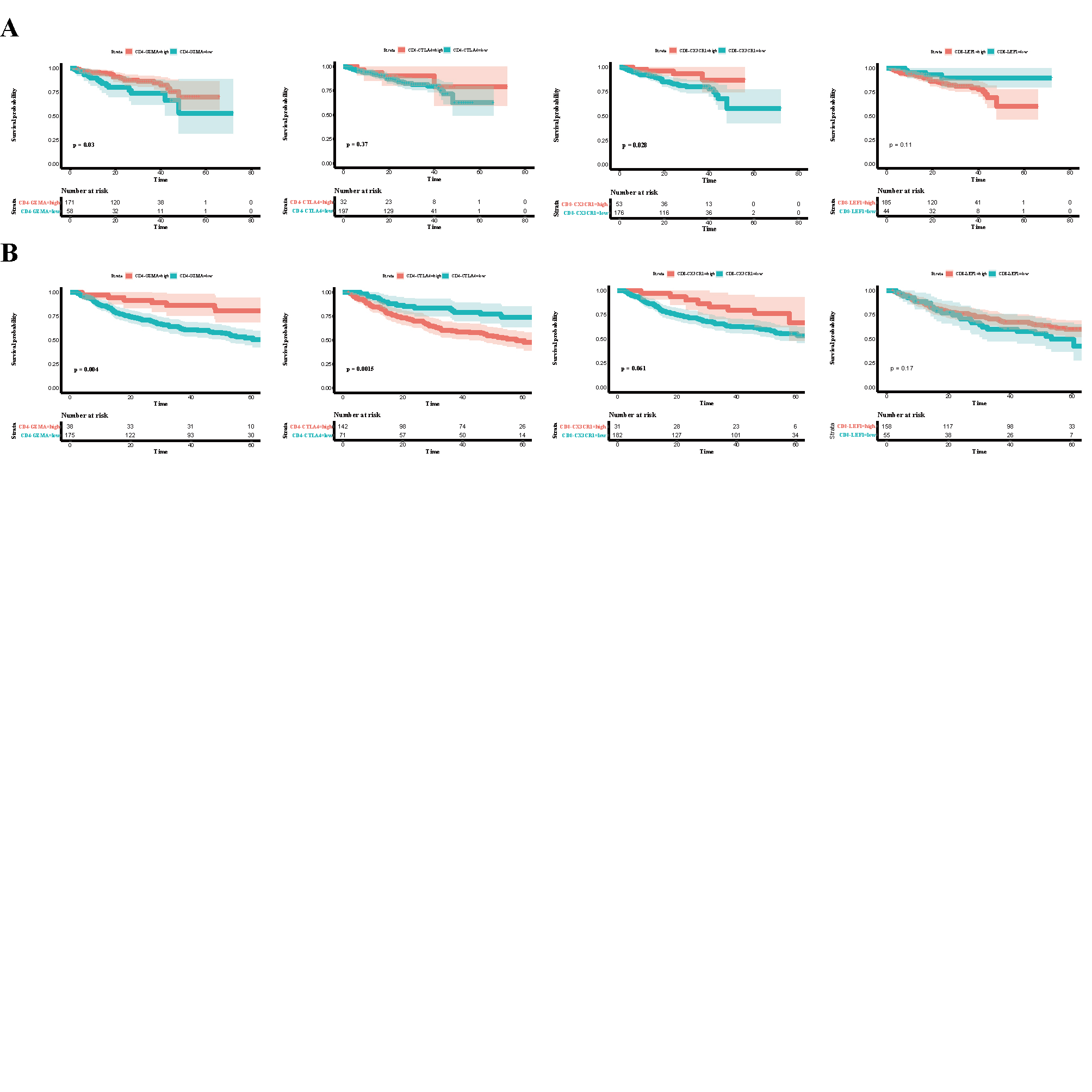

Supplement: Supplementary Figure 2 — Association between survival and four immune cell subsets in ICGC and GSE14520 cohorts. (A,B) Survival plots of immune cell subsets (CD4-GZMA, CD4-CTLA4, CD8-CX3CR1, CD8-LEF1) in ICGC (A) and GSE14520 (B) cohorts. Time was calculated by month. Depicted p-values are from log-rank tests. [file Image_2.JPEG]

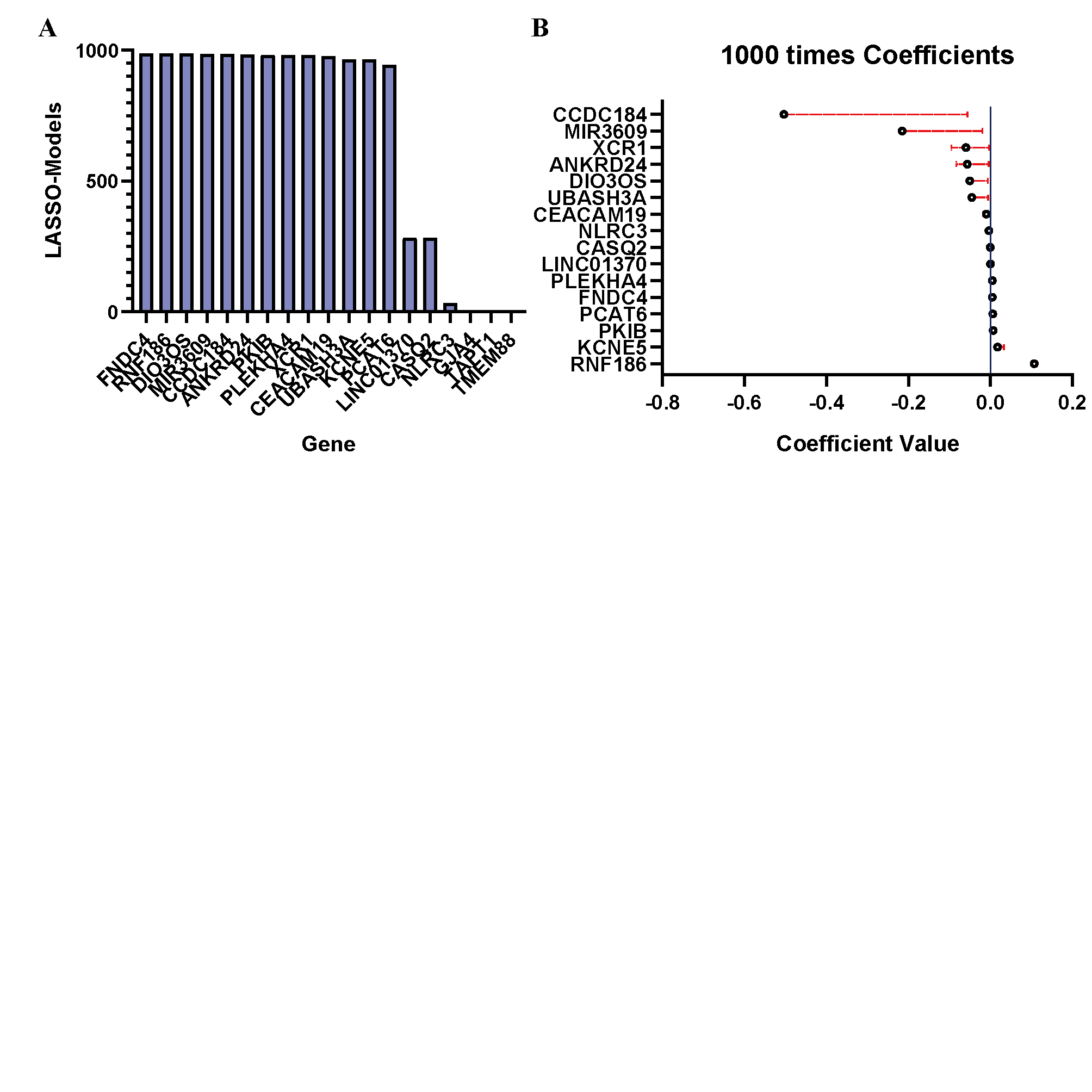

Supplement: Supplementary Figure 3 — The frequency and coefficients of representative genes during 1,000 iterations of LASSO Cox regression. (A) Frequency of representative genes investigated in 1,000 iterations of LASSO Cox regression. (B) Box plot for mean and confidence intervals of coefficients. [file Image_3.JPEG]

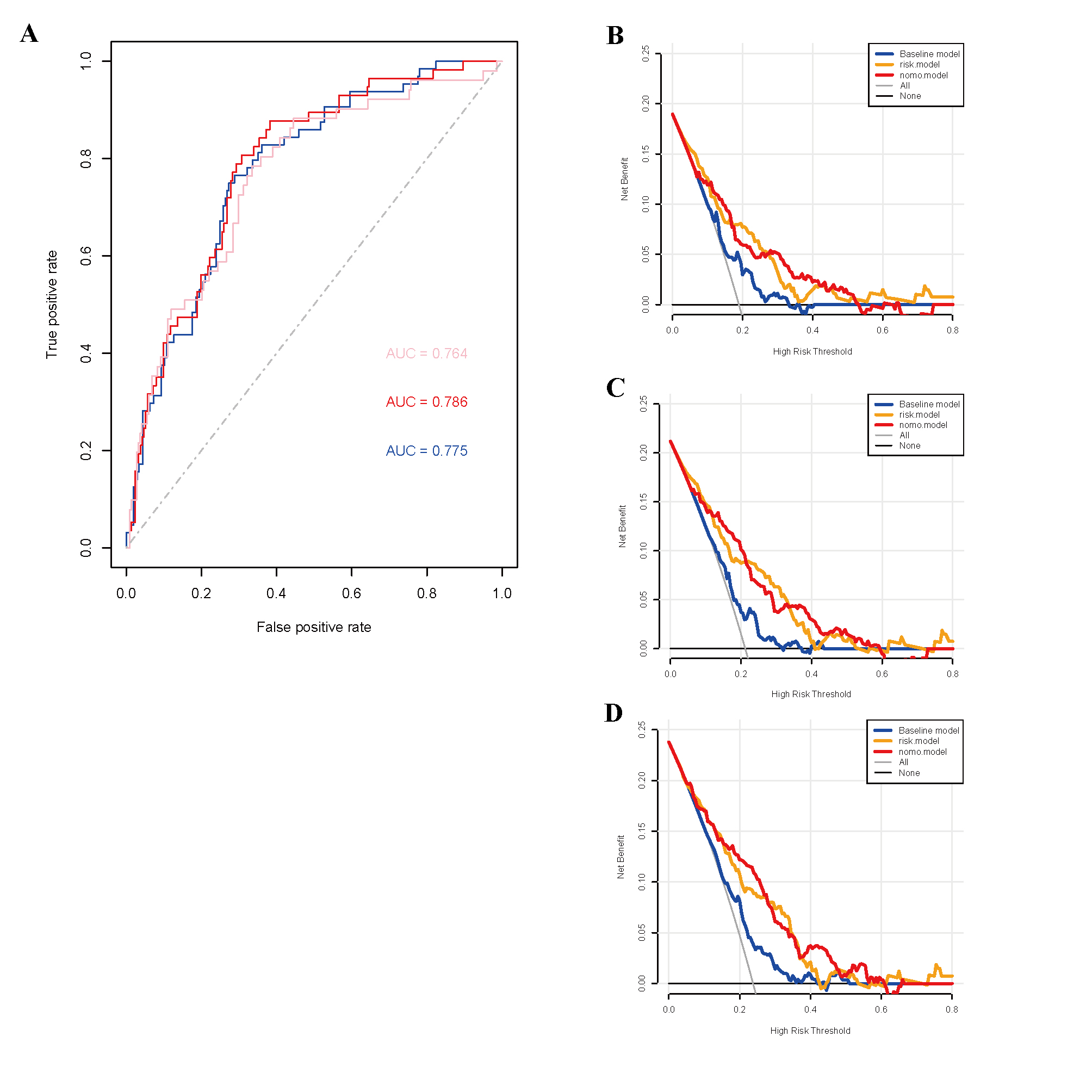

Supplement: Supplementary Figure 4 — The evaluation of the performance of the nomogram from TCGA cohort in predicting OS in different years. (A) Time-dependent ROC curve of the nomogram. The area under the ROC curve were 0.764, 0.786, and 0.775 for the risk score at 3, 4, and 5 years, respectively. (B–D) The Decision Curve Analysis (DCA) of the nomogram. [file Image_4.JPEG]

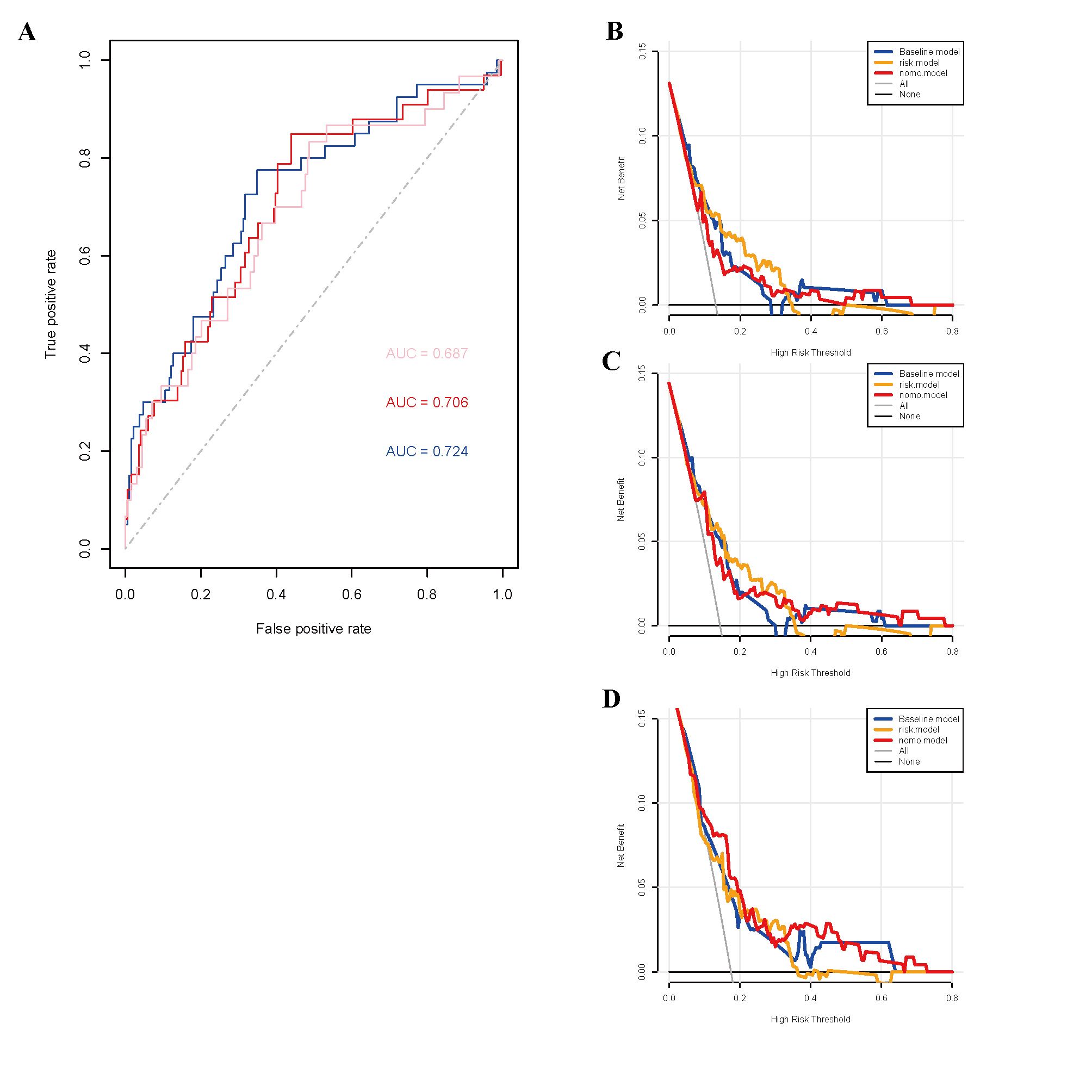

Supplement: Supplementary Figure 5 — The evaluation of the performance of nomogram from ICGC cohort in predicting OS in different years. (A) Time-dependent ROC curve of the nomogram. The area under the ROC curve were 0.764, 0.786, and 0.775 for the risk score at 2, 3, and 4 years, respectively. (B–D) The Decision Curve Analysis (DCA) of the nomogram. [file Image_5.JPEG]
